# Sugar Profile, Mineral Content, and Rheological and Thermal Properties of an Isomerized Sweet Potato Starch Syrup

**DOI:** 10.1155/2013/243412

**Published:** 2013-12-24

**Authors:** Brunson Dominque, Peter N. Gichuhi, Vijay Rangari, Adelia C. Bovell-Benjamin

**Affiliations:** ^1^Department of Food and Nutritional Sciences, Tuskegee University, 300-A Campbell Hall, Tuskegee, AL 36088, USA; ^2^Center for Advanced Material Science Testing, Tuskegee University, Tuskegee, AL 36088, USA

## Abstract

Currently, corn is used to produce more than 85% of the world's high fructose syrup (HFS). There is a search for alternative HFS substrates because of increased food demand and shrinking economies, especially in the developing world. The sweet potato is a feasible, alternative raw material. This study isomerized a high glucose sweet potato starch syrup (SPSS) and determined its sugar profile, mineral content, and rheological and thermal properties. Rheological and thermal properties were measured using a rheometer and DSC, respectively. Sweet potato starch was hydrolyzed to syrup with a mean fructose content of 7.6 ± 0.4%. The SPSS had significantly higher (*P* < 0.05) mineral content when compared to commercial ginger and pancake syrups. During 70 days of storage, the SPSS acted as a non-Newtonian, shear-thinning liquid in which the viscosity decreased as shear stress increased. Water loss temperature of the SPSS continually decreased during storage, while pancake and ginger syrups' peak water loss temperature decreased initially and then increased. Further and more detailed studies should be designed to further enhance the fructose content of the syrup and observe its stability beyond 70 days. The SPSS has the potential to be used in human food systems in space and on Earth.

## 1. Introduction

The National Aeronautics and Space Administration (NASA) has partnered with scientists at the Center for Food and Environmental Systems for Human Exploration of Space (CFESH) at Tuskegee University to incorporate the sweet potato as a viable dietary item in the space food system. According to NASA, foods in the space system are designed to supply the recommended dietary allowances of the essential nutrients to perform in the space environment. The overall objective of the Food Processing and Product Development Team (FPD) of CFESH is to convert raw crops such as sweet potatoes into ingredients and nutritious value-added food products for use in space food systems, with applications for consumers on Earth. Value-added products developed by FPD include a ready-to-eat sweet potato breakfast cereal [1, 2], a sweet potato bread [3, 4], a sweet potato starch syrup [5, 6], and a sweet potato beverage [7].

Sweet potato (*Ipomoea batatas* (L.) Lam.) is a popular root crop in the United States (USA) and developing countries, which contains roughly 16 to 24% starch [8]. Starches play a vital role in food product development, for example, as raw material, additives, or texture enhancers [9]. Recently, the sweet potato has been increasingly used as an industrial crop for the production of glucose and high fructose syrup [10]. FPD has used sweet potatoes as a source of starch for syrup production; although a consumer acceptable high glucose syrup has been developed, its storage stability needs to be further improved. A feasible alternative for such improvement is the production of an isomerized fructose syrup due to its noncrystalline nature, ability to prevent microbial growth and extend shelf life. Additionally, fructose is sweeter than sucrose—in comparative studies of sweetness, in which the sweetness of sucrose was set at 100, fructose had a sweetness of 173, and glucose had a sweetness of 74.

Gore et al. [11] reported the commercial manufacture of sweet potato syrup, with the possibilities for use as table syrup, for baking and cooking purposes, and for blending with other syrups to prevent crystallization. At CFESH, Silayo et al. [12] developed an engineering system for converting sweet potato into glucose syrup. Concentration trials utilizing the system produced syrups with volumes of 100 and 70 mL with the respective dextrose equivalence of 281 and 213 mg/mL. Johnson et al. [13] investigated the feasibility of using native cassava/sweet potato flours and their blends with rice flour and wheat flour, as the raw material for high fructose syrup production. They reported that cassava/sweet potato or their blends with cereal flours had higher fructose yields than native cassava flour and cassava-rice blends.

Food products such as syrups contain ingredients that have a major impact on the rheology of the final product [14]. Although researchers have reported the development of sweet potato syrups, very few, if any, have examined its rheological properties. Knowledge of the rheological characteristics of the sweet potato starch syrup (SPSS) is valuable in predicting pourability and the ease with which it may be handled, processed, or used. Rheological factors play an important role in consumers' selection and continued use of a syrup [15]. Products such as pancake syrup which appear too thick or too runny have little appeal to consumers [16].

Data regarding the effect of storage on the rheological and thermal behaviors of an isomerized SPSS are limited. This is one of few, if any, studies to report on the rheological and thermal properties of a SPSS during storage. The study was part of the larger CFESH and FPD project to convert raw crops such as sweet potatoes into ingredients and nutritious value-added food products for use in food systems in space and on Earth. The overall objective of this study was to improve the quality and storage stability of an isomerized SPSS. Specifically, the study isomerized a high glucose SPSS and determined its sugar profile, mineral content, and rheological and thermal properties.

## 2. Materials and Methods

### 2.1. Raw Materials

Sweet potatoes (variety: Mississippi Red) purchased from the local grocery store were utilized to develop the SPSS. Sweet potatoes were washed free of dirt, weighed, hand-peeled, and shredded. Our laboratory has utilized other varieties of sweet potato in syrup production with good outcomes. For example, earlier studies by Miller (2003) and Ibrahim (2004) utilized Hillbilly and Whatley/Loretan (hydroponic and field-grown) sweet potatoes in syrup production. The variety or varieties used are usually reliant on University farm production.

### 2.2. Isolation of Sweet Potato Starch

Sweet potato starch was isolated following the procedures described by Miller [5]. Briefly, the shredded roots were weighed and homogenized at 1 : 1 water/sweet potato ratio. The resulting puree was centrifuged at 3300 rpm for 5 minutes. The supernatant was then decanted and all precipitates collected. The sediments were dehydrated for 12 h at 70°C. The resulting starch was ground in a laboratory blender at high speed for 30 seconds. After grinding, the starch was milled, vacuum-sealed, and stored at room temperature (22 ± 2°C) until further use.

### 2.3. Sweet Potato Starch Syrup Production

#### 2.3.1. Liquefaction

Food-grade enzymes, Spezyme FRED (thermostable *α*-amylase), Optidex L-400 (glucoamylase), Gensweet SGI (glucose isomerase), and Optimax L-1000 (pullulanase), were supplied by courtesy of Genencor Danisco Division (Rochester, New York, USA). The enzymes had the following activities: Spezyme FRED an activity of ≥17,400 LU/g (LU is liquefon units); Optidex L-400 a minimum activity of 350 GAU/g (glucoamylase unit); and Optimax L-1000 an activity of 1000–1260 ASPU/g (acid stable pullulanase units).

SPSS was produced following the procedures described by Miller [5] and Ibrahim [6]. Sweet potato starch (30 g) was mixed with 400 mL distilled water, and the solution was heated to 100°C to promote gelatinization of the starch. The pH of the starch was adjusted to 4.5 with NaOH or HCl as needed. Upon gelatinization, *α*-amylase (4.5 mL) was added to the solution to promote liquefaction. The solution was then incubated at 90°C for 2 hours and allowed to cool to 62°C.

#### 2.3.2. Saccharification

The cooled solution was treated with 300 *μ*L glucoamylase and 300 *μ*L pullulanase, incubated at 62°C for 12 h to induce glucose formation from the liquefied starch. After incubation, the solution was vacuum-filtered.

#### 2.3.3. Isomerization

The pH of the solution was adjusted to 7.5 and 200 *μ*L glucose isomerase was added. The solution was then kept in a thermostatic water bath at 60°C for 5 h, after which it was concentrated to 63−73.9°Brix. The syrup was measured, bottled, labeled, and stored at room temperature (22 ± 2°C) for further evaluation.

#### 2.3.4. Samples

The samples evaluated were the isomerized SPSS and two commercial syrups (pancake and ginger). Karo brand pancake (corn) syrup was purchased from a local grocery store, and ginger syrup was provided by courtesy of Buderim Ginger (Mahwah, New Jersey, USA).

#### 2.3.5. Sugar Profile

The sugar profile of the syrups was tested at Medallion Labs (Minneapolis, Minnesota, USA). Fructose, glucose, sucrose, maltose, and lactose were determined. The sugars were extracted, diluted, and quantitated using High Performance Liquid Chromatography (HPLC).

#### 2.3.6. Mineral Content

Calcium, magnesium, phosphorus, iron, and potassium contents of the syrups were tested at Medallion Labs (Minneapolis, Minnesota, USA). Samples were ashed and then digested in acid to solubilize metal ions. The digests were quantitatively added to a known volume of acid. Inductively coupled plasma (argon plasma) was done on the solutions and quantitations were provided by a standard curve. Iron and potassium were measured using atomic absorption.

#### 2.3.7. Rheological Measures

Aliquots (10 mL) of the SPSS were stored in plastic bottles, sealed, and stored at room temperature (22 ± 2°C) for 70 days. Samples were randomly withdrawn for testing on Days 0, 15, 30, 49, and 70, and rheological measures were conducted. However, data from Day 15 were excluded from the analysis because of technological difficulties with the equipment that day, and data were considered unreliable. Commercially available pancake and ginger syrups were also tested.

#### 2.3.8. Instrumental

*Viscosity versus Shear Rate, and Shear Stress*. Steady viscosity, shear rate and shear stress testing were conducted using the TA Instruments AR-2000 rheometer (New Castle, Delaware USA) and modifications of the procedures were described by Ngadi and Yu [17]. Tests of viscosity versus shear rate and viscosity versus shear stress as a function of time were conducted at a fixed temperature (25°C), and the shear rate and shear stress were varied. The rheometer was calibrated to 1000 *μ*m minimum gap space; the syrup samples were spread across the bottom plate, and the gap height was set at 4000 *μ*m. Measurements were conducted in duplicate.

#### 2.3.9. Subjective

*Line Spread Test (LST)*. SPSS, pancake, and ginger syrups were tested using the methods described by Budke et al. [18]. A hard clear plastic surface was marked with concentric circles spaced 0.5 cm apart from a 2.5 to 7.5 cm radius. Lines radiating from the center intersected the circles and divided them into four quadrants. The syrup samples were held in a hollow cylinder positioned at the center of the circles for a setting time of 10 minutes. The cylinder was then lifted to allow the sample to spread for 60 seconds. The mean measurement, averaged across bisecting lines at the four quadrants, represented the degree of thickness of the syrup. LST was conducted on SPSS, pancake, and ginger syrup on the same days as the instrumental tests were conducted.

#### 2.3.10. Thermal Measures

*Differential Scanning Calorimetry (DSC)*. Thermal scanning of the syrup samples was carried out using the Mettler DSC 822e (Columbus, Ohio, USA), equipped with a refrigerated cooling system that efficiently controlled and monitored temperature up to 250°C. Approximately 2–5 mg of the syrup samples was weighed into aluminum pans, which were hermitically sealed and placed in the DSC. Scans were conducted at 5°C/min from 30°C to 250°C. Water loss temperatures were recorded.

#### 2.3.11. Statistical Analysis

One-way analysis of variance (ANOVA) at a ≤0.05 level of probability was used as the criterion of significance for the sugar profile and mineral content tests. Repeated measures ANOVAs were used to (1) compare the viscosity measures (instrumental and LST) of the SPSS, pancake, and ginger syrups throughout storage and (2) compare the thermal measurements of SPSS, pancake, and ginger syrups to determine whether there were differences among the syrup samples during storage, and Fisher's LSD (*P* < 0.05) was used to determine where those differences lie. Pearson correlations were used to assess the nature of the relationship between the instrumental and LST measures.

## 3. Results and Discussion

### 3.1. Sugar Profile of the Syrups

**Figure 1:**
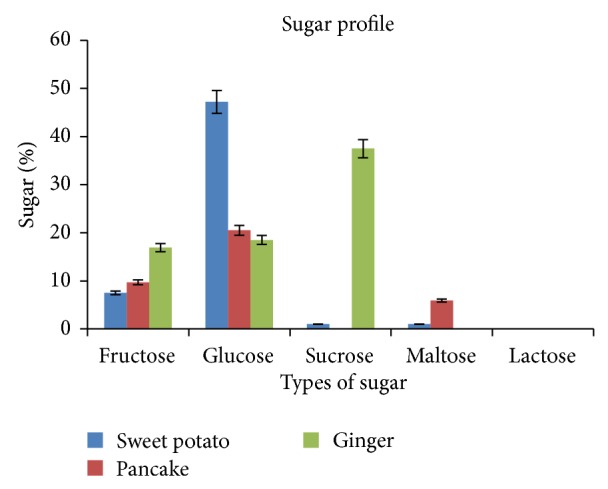
Sugar profile of sweet potato starch, pancake, and ginger syrups.

The starch content of the sweet potato was roughly 81% and the ratio 14–7% amylose: 86–93% amylopectin ratio. The sugar profiles of the syrups are shown in Figure 1. The average amount of fructose in the SPSS was 7.6 ± 0.4%. The glucose syrup produced from the SPSS was concentrated to 63–73.9°Brix and was then isomerized at 60°C for 5 h using glucose isomerase. Prior to isomerization, the SPSS had a glucose content of 51% and <1.0% fructose. The intention was to improve the fructose content by extension of the sweetness and storage stability of the SPSS through isomerization. After isomerization, although the fructose yield was slightly improved, the major sugar found in the SPSS was glucose (47.2 ± 1.8%). Perhaps, in this study, the incubation time was too short for isomerization of glucose to fructose. Our study used 60°C and 5 h incubation time, whereas Johnson et al. (2009) [10] used 60°C and 48 h incubation time for isomerization to a high fructose sweet potato syrup. It should be noted that the fructose yield in the current study was higher (7.6 or 7.6/100 g versus 5.7 or 5.7/100 g) than Johnson et al.'s [10], who used direct conversion of sweet potato roots and not the isolated starch. Furthermore, older studies [19] reported that the enzyme requires magnesium and cobalt as activators; neither of these were used in the current study.

### 3.2. Mineral Content of the Syrups

The SPSS had higher amounts of calcium than the pancake and ginger syrups (Table 1). It has been reported by Crabb and Shetty [20] that it is preferable to remove calcium ions prior to isomerization because they inhibit the isomerization of glucose into fructose. The possible inhibition of glucose isomerase by calcium ions may be due to the competition with the required magnesium ions for the enzyme's active site [21]. It has been reported by Kasumi et al. [22] that the enzyme glucose isomerase depends on either magnesium or manganese ion for its activity to take place. It is speculated that the Ca content of the SPSS negatively affected the isomerization process of glucose to fructose in the syrup.

Table 1 shows the mineral contents of the syrups. The mean calcium content (32.9 ± 0.3 mg/100 g) of the SPSS was significantly (*P* < 0.05) higher than that of the pancake and ginger syrups. The calcium content was roughly eight and 16 times more in the SPSS than that in the ginger and pancake syrups, respectively. The high calcium content of the SPSS could have been due to the typically high calcium content of sweet potatoes, although calcium content of different varieties of sweet potatoes could vary greatly [23].

The average amount of magnesium in the SPSS was 23.4 ± 0.6 mg/100 g (Table 1). Similar to the calcium, the magnesium content of the SPSS was significantly (*P* < 0.05) higher than the two other syrups, which had negligible amounts of magnesium. The pattern was similar for phosphorus; the SPSS had a significantly (*P* < 0.05) higher phosphorus content (49.7 ± 0.1 mg/100 g) than the pancake and ginger syrup (Table 1). All three syrups had low iron contents. The iron contents of SPSS and ginger syrup were similar with an average of 0.5 ± 0.0 and 0.6 ± 0.0 mg/100 g, respectively, but the pancake syrup had significantly (*P* < 0.05) less iron (Table 1). Likewise, the mean potassium content of the SPSS was 268 ± 8.5 mg/100 g (Table 1), which was significantly (*P* < 0.05) higher and several times more than that of the pancake and ginger syrups. The ginger and pancake syrups had similar potassium contents. Overall, the results of this study demonstrated that the SPSS had higher mineral (calcium, magnesium, phosphorus, iron, and potassium) content when compared to the ginger and pancake syrups.

### 3.3. Rheological Measures of Syrups

In general, as shear rate increased the apparent viscosity of the SPSS, pancake, and ginger syrups decreased, which resulted in the syrups exhibiting shear-thinning behavior as described by Abu-Jdayil et al. [24]. These shear-thinning effects indicated that the SPSS and other syrups behaved as non-Newtonian fluids. In shear-thinning fluids, viscosity decreases or increases as the applied shear stress increases [14]. The shear-thinning behavior observed in the three syrup samples could be attributed to stress-induced breakdown of the network structure [24]. Similar to Herh et al. [14] and Abu-Jdayil et al. [24], the phenomenon of shear thickening was not observed in the syrup samples used in the current study.

**Figure 2:**
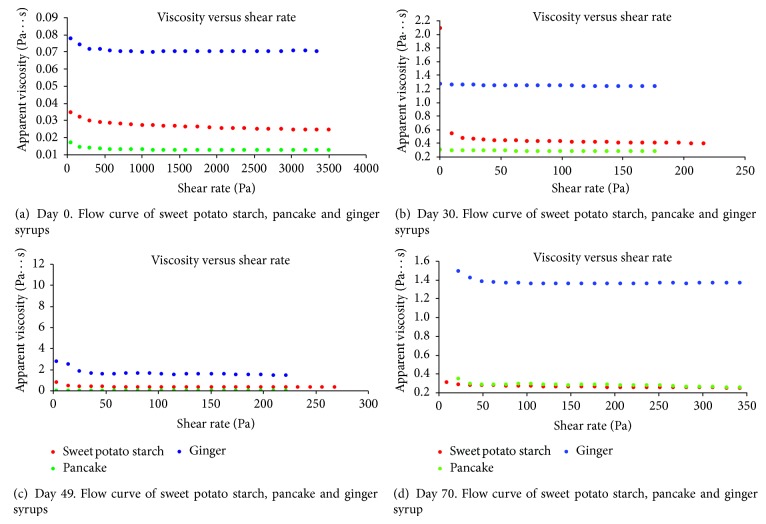
Viscosity versus shear rate for the SPSS, pancake, and ginger syrups at (a) Day 0, (b) Day 30, (c) Day 49, and (d) Day 70.

The flow curves of the SPSS, pancake and ginger syrups are shown in Figures 2(a)–2(d). It can be seen in Figure 2(a) that, after a sharp reduction, the viscosity was stabilized at high shear rates. This result suggests that the onset of shear thinning occurs at a lower shear rate. It has been speculated that this could be due to the onset of entanglements caused by the presence of the highly branched, high molecular weight amylopectin [25]. Sweet potatoes contain high amounts of the branched-chain polymer amylopectin, with branch points occurring through *α*-1-6 bonds. Generally, amylopectins occur in most cereals and plants, contributing to about 80 to 85% of the total starch content [26].

According to Kelco [27], high viscosity is desirable only under low shear rate conditions and should decrease as flow rate increases. SPSS, pancake, and ginger syrup had their highest viscosities under the lowest shear rates, and viscosity of the syrups decreased as flow increased. Viscosity readings of the three syrups were observed at 65 seconds for each trial. On Day 0, there were no significant differences among the viscosities of the three syrups. However, on Days 30, 49, and 70, viscosities of the syrups were significantly (*P* < 0.05) different. On Day 30, all the three syrups were significantly (*P* < 0.05) different from one another.

The pancake and ginger syrups had similar viscosities on Days 49 and 70 but were significantly (*P* < 0.05) different from the SPSS. The mean viscosity of the SPSS, pancake, and ginger syrup increased between Days 0 and 30 and decreased between Days 49 and 70. The decrease in viscosity implies a progressive breakdown of the syrups' structure [28]. Strain is imposed on syrup when it is poured into bottles for storage and also when the syrup is poured unto waffles or pancakes by consumers; therefore data from this study confirmed that the viscosity of SPSS, pancake, and ginger changed over time as strain was applied.

**Figure 3:**
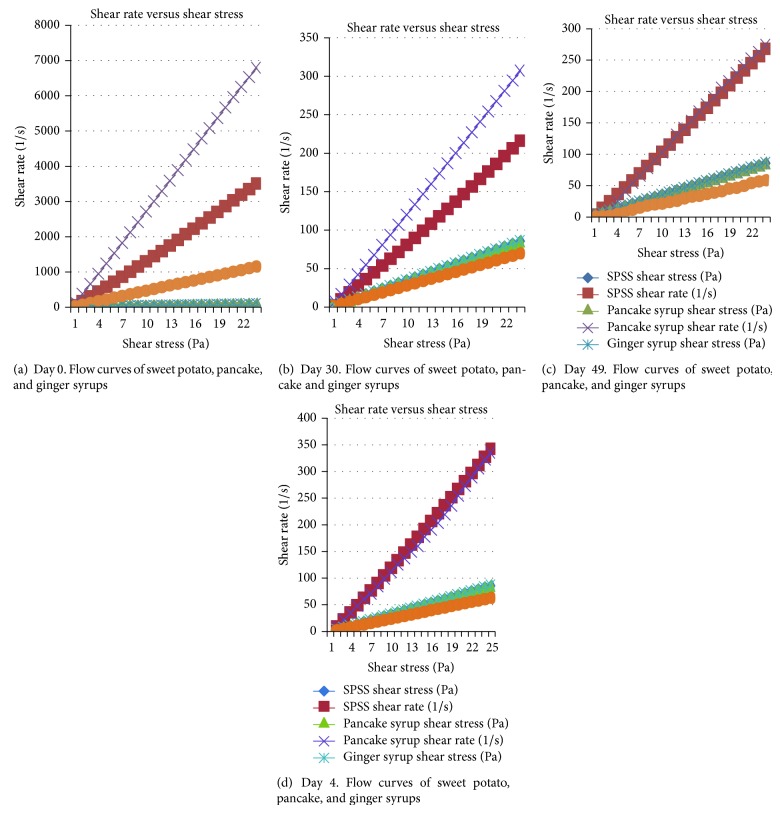
Flow curves for sweet potato starch, pancake, and ginger syrups.

Typical shear rates versus shear stress responses of the SPSS, pancake, and ginger syrups at the constant temperature of 22°C are shown in Figures 3(a)–3(d). As expected, shear stress increased with increasing rate in a fairly linear fashion. The mean apparent viscosity ranged from 0.03 to 0.50, 0.01 to 14.2, and 0.07 to 2.2 Pa·s for SPSS, pancake, and ginger syrups, respectively, during storage. In general, scarce information is available in the published literature regarding the rheology of sweet potato syrup. However, Ngadi and Yu [17] reported apparent viscosities ranging from 0.035 to 0.651 Pa·s for different grades and temperatures of maple syrups. Johnson [29] also reported the viscosity of maple syrup at 25°C as 0.1635 Pa·s. The viscosity for SPSS at 25°C (0.03 to 0.5 Pa·s) in the current study was consistent with the range obtained for maple syrup.

On Days 0, 30, and 70, ginger syrup had the highest viscosity while, on Day 49, the viscosity of pancake syrup was highest. Compared to the results for Days 0, 30, and 70, the pancake syrup displayed unusual elevations in viscosity on Day 49, even though extreme care was taken while conducting the experiment and measures were done in duplicate with the intent of minimizing errors. It is still possible that, despite the care taken, some type of experimental error influenced the result for the pancake syrup on Day 49. Overall, the SPSS and pancake syrup were the thinner syrups with similar viscosities on Days 0 and 70.

### 3.4. Line Spread Test (LST)

Viscosity readings of the SPSS using LST were similar to the instrumental rheological data. As with the instrumental data, on Day 0, the viscosities of the three syrups were similar. Pancake and ginger syrup had a significantly (*P* < 0.05) higher viscosity than SPSS on Day 30. On Days 49 and 70, pancake and ginger syrups were similar to each other.

At the beginning of the storage study there was a weak positive relationship (*r* = 0.4) between the line spread and instrumental measures. However, on Days 30, 49, and 70, the relationship between the line spread and instrumental measures was strong (*r* = 0.8, *r* = 0.7, and *r* = 0.9, resp.). It is unclear why the results from Day 0 of line spread and instrumental measures were poorly correlated. We can only speculate that the viscosities of the syrups were not yet stabilized on Day 0, which resulted in its poor correlation.

### 3.5. Differential Scanning Calorimetry (DSC)

**Figure 4:**
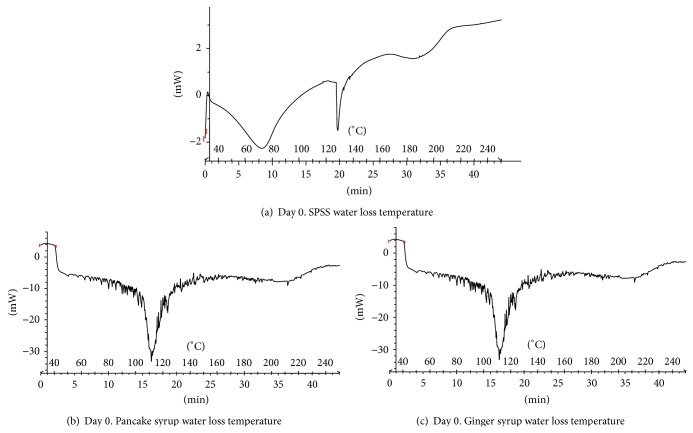
Water loss temperature of sweet potato, pancake and ginger syrups on Day 0 of storage.

The water loss curves obtained during DSC scanning of the SPSS, pancake, and ginger syrup samples from Day 0 are presented in Figure 4. Each syrup sample showed an endothermic curve which indicates that the syrups absorbed energy during heating. Water loss temperatures and melting endotherms of syrups are fairly broad because sugars do not have sharp water loss temperatures and their water loss proceeds over a temperature range. The melting temperatures of sugars are sensitive to water, impurities, and crystallinity [30].

Water loss temperatures were recorded as the transition peak, which were found to vary among the syrup samples from day to day. Water loss temperature of the SPSS increased from Days 0 to 30 and then decreased from Days 30 to 70. Pancake and ginger syrups' peak water loss temperature decreased from Days 0 to 49 and then increased from Days 49 to 70 (Table 2). Sugar concentrations of the syrups could reflect the water loss temperature.

As stated previously, glucose was the predominant sugar in the SPSS. The increase in water loss temperature from Days 49 to 70 for pancake and ginger syrups could be related to the fact that they had higher fructose contents than the SPSS. Ginger syrup had the highest water loss temperature of all syrups and also the highest percentage of fructose.

The water loss temperatures of SPSS, pancake, and ginger syrups were similar during the storage study. This result indicates that the water loss temperature of the SPSS was similar to the commercial pancake and ginger syrups. This study lasted for only 70 days and 60°C was the lowest recorded water loss temperature for SPSS. It would require a longer time study to dictate whether or not the water loss temperature goes below 60°C.

### 3.6. Economic Feasibility and Sensory Acceptability

The present study utilized the batch process for syrup production because of available laboratory equipment. In the production of a corn-based high fructose syrup (HFS), a continuous process is usually used, which is more time and energy efficient than the batch process. However, if the continuous process is employed for the production of SPSS, the authors believe that it will be much more economically feasible than the corn-based HFS. In terms of sensory analysis, Ibrahim (2004) determined the acceptability of the sweet potato syrup in 112 sixth grade students. The SPSS was acceptable to the sixth graders. The isomerized SPSS has been tested in college-age students; the dataset is being analyzed.

## 4. Conclusion

Thermostable *α*-amylase, glucoamylase, glucose isomerase, and pullulanase were used to hydrolyze sweet potato starch into syrup with comparable, and in some instances superior nutritional, rheological and thermal properties to commercially available ginger and pancake syrups using the enzymes. Our study has added important information to the knowledge base regarding the rheological and thermal properties of the SPSS. Additionally, the SPSS had significantly higher mineral content than the ginger and pancake syrups, making it a much more nutritious option. Further and more detailed studies should be designed to further enhance the fructose content of the syrup and observe its stability beyond 70 days. Overall, the SPSS has the potential to be used in food systems in space and on Earth.

## Figures and Tables

**Table 1:** Mean mineral content of sweet potato starch, pancake, and ginger syrups.

Types of syrup	Calcium (mg/100 g)	Magnesium (mg/100 g)	Phosphorus (mg/100 g)	Iron (mg/100 g)	Potassium (mg/100 g)
Sweet potato starch	32.9 ± 0.3^a^	23.4 ± 0.6^a^	49.7 ± 0.1^a^	0.5 ± 0.0^a^	268 ± 8.5^a^
Pancake	1.2 ± 0.0^c^	0.1 ± 0.0^c^	2.5 ± 0.1^b^	0.1 ± 0.1^b^	12.0 ± 4.0^b^
Ginger	4.4 ± 0.0^b^	2.3 ± 0.0^b^	0.5 ± 0.0^c^	0.6 ± 0.0^a^	2.4 ± 1.9^b^

Means with the same letters in each column are not significantly different (*P* < 0.05).

**Table 2:** Water loss temperatures of SPSS, pancake, and ginger syrups.

Types of syrup	Water loss temperature (Day 0)	Water loss temperature (Day 30)	Water loss temperature (Day 49)	Water loss temperature (Day 70)
Sweet potato starch	70°C	88°C	80°C	60°C
Pancake syrup	110°C	95°C	73°C	90°C
Ginger syrup	130°C	130°C	120°C	140°C
